# Sevoflurane inhibits progression of glioma via regulating the HMMR antisense RNA 1/microRNA-7/cyclin dependent kinase 4 axis

**DOI:** 10.1080/21655979.2021.1976712

**Published:** 2021-10-30

**Authors:** Xi’an Bao, Yibo Peng, Jun Shen, Longqiu Yang

**Affiliations:** a Department of Anesthesiology, The Affiliated Nanchang Hospital of SUN YAT-SEN University, Nanchang, 330006, China; b Department of Anesthesiology, Chinese Medicine Hospital of Yangxin County, Huangshi, China; c Department of Anesthesiology, Huangshi Central Hospital, Affiliated Hospital of Hubei Polytechnic University, Edong Healthcare Group, Huangshi, China; d Medical College, Wuhan University of Science and Technology, Wuhan, China

**Keywords:** Glioma, sevoflurane, HMMR-AS1, miR-7, CDK4

## Abstract

Sevoflurane (Sev) is a volatile anesthetic that can inhibit tumor malignancy. Glioma is a main brain problem, but the mechanism of Sev in glioma progression is largely unclear. This study aims to explore a potential regulatory network of long noncoding RNA (lncRNA)/microRNA (miRNA)/mRNA associated with the function of Sev in glioma progression. LncRNA HMMR antisense RNA 1 (HMMR-AS1), miR-7 and cyclin-dependent kinase 4 (CDK4) abundances were examined via quantitative reverse transcription polymerase chain reaction and western blot. Cell viability, invasion, and colony formation ability were analyzed via cell counting kit-8, transwell analysis, and colony formation. The target association was analyzed via dual-luciferase reporter analysis and RNA pull-down. The *in vivo* function of Sev was investigated by xenograft model. HMMR-AS1 abundance was increased in glioma tissues and cells, and reduced via Sev. Sev constrained cell viability, invasion, and colony formation ability via decreasing HMMR-AS1 in glioma cells. miR-7 expression was decreased in glioma, and was targeted via HMMR-AS1. HMMR-AS1 silence restrained cell viability, invasion, and colony formation ability by up-regulating miR-7 in glioma cells. Sev increases miR-7 abundance via decreasing HMMR-AS1. CDK4 was targeted via miR-7, and highly expressed in glioma. miR-7 overexpression inhibited cell viability, invasion, and colony formation ability via reducing CDK4 in glioma cells. CDK4 expression was reduced by Sev via HMMR-AS1/miR-7 axis. Sev suppressed cell growth in glioma by regulating HMMR-AS1. Sev represses glioma cell progression by regulating HMMR-AS1/miR-7/CDK4 axis.

## Introduction

Glioma is the most common central nervous system malignancy with high recurrence and poor prognosis [1]. The use of anesthetic gases provides potential strategies for glioma treatment [2]. Sevoflurane (Sev) is a widely used anesthetic gas by interacting with γ-aminobutyric acid [3]. Multiple reports suggest Sev has an anti-glioma activity by decreasing cell proliferation, migration and invasion [4–6]. However, the mechanism underlying Sev in glioma treatment remains poorly understood.

Noncoding RNAs participate in glioma initiation and progression [7]. Long noncoding RNAs (lncRNAs; >200 nucleotides) play crucial roles in glioma progression by regulating microRNA (miRNA)/mRNA axis [8]. For example, lncRNA ZFPM2 antisense RNA 1 promotes glioma progression by regulating the miR-515-5p/superoxide dismutase 2 [9]. Furthermore, lncRNAs are associated with Sev-mediated neuronal toxicity [10]. LncRNA HMMR antisense RNA 1 (HMMR-AS1) can promote the progression of human tumors, like breast cancer, ovarian cancer and lung adenocarcinoma [11–13]. Moreover, HMMR-AS1 knockdown represses glioma growth, migration and invasion [14]. Nevertheless, it is unclear whether HMMR-AS1 can participate in the anti-glioma function of Sev.

The differential expressions of miRNAs (~22 nucleotides) are related to brain tumors, which are associated with glioma progression and treatment [15,16]. Previous studies report miR-7 can repress cell growth, migration, and invasion in glioma via regulating insulin-like growth factor 1 receptor and trefoil Factor 3 [17,18]. But it is uncertain whether miR-7 is interacted with HMMR-AS1. The cyclin-dependent kinases (CDKs) are important biomarkers for tumor treatment [19]. CDK4 is a key member in CDKs, which is relevant to human tumor progression [20]. Moreover, CDK4 contributes to glioma cell growth, which is mediated via miR-129 [21]. However, whether CDK4 is respond to Sev, and whether it is mediated by HMMR-AS1 and miR-7 are unknown.

The novelty of the present work is to explore new mechanism targeted by Sev in glioma progression. Here we hypothesized that Sev might target the HMMR-AS1/miR-7/CDK4 axis to regulate glioma progression. The purposes of our research were to explore the role of Sev in glioma cell viability, invasion, and colony formation ability, and analyze the interaction between Sev and the HMMR-AS1/miR-7/CDK4 axis in glioma cells. This study may provide a new insight into the effect of Sev on glioma treatment.

## Methods and materials

### Bioinformatics analysis

The top 10 genes including CDK4 in glioma was explored according to Gene Expression Profiling Interactive Analysis (GEPIA) database (http://gepia.cancer-pku.cn/) [22], and related information was displayed in Table 1.The targets of HMMR-AS1 were searched via LncBase V.2 (http://carolina.imis.athena-innovation.gr/diana_tools/web/index.php?r= lncbasev2/index) [23]. The targets of miR-7 were predicted by microT-CDS (http://diana.imis.athena-innovation.gr/DianaTools/index.php?r=microT_CDS /index) [24].

**Table 1.** The expression information of top 10 up-regulated genes in glioma via GEPIA

Gene Symbol	Gene ID	Median (Tumor)	Median (Normal)	Log2(Fold Change)	Percentage
CDK4	ENSG00000135446.16	4522.327	240.059	4.23	1.00E+0
SEC61G	ENSG00000132432.13	10,997.591	594.692	4.207	1.00E+0
TSPAN31	ENSG00000135452.9	1874.383	100.586	4.206	1.00E+0
RP11-698N11.2	ENSG00000254919.1	33.864	1.045	4.092	1.00E+0
LANCL2	ENSG00000132434.9	544.089	43.334	3.62	1.00E+0
MSMP	ENSG00000215183.4	113.379	8.994	3.517	9.38E-1
DLL3	ENSG00000090932.10	151.675	12.349	3.516	1.00E+0
MAR9	ENSG00000139266.5	671.781	61.226	3.435	1.00E+0
EGFR	ENSG00000146648.15	1052.862	102.515	3.348	1.00E+0
RP11-231 C18.1	ENSG00000248184.1	70.484	6.019	3.348	1.00E+0

## Patient tissues

The glioma tissues were collected from 37 glioblastoma patients (17 cases at WHO grades I and II, and 20 cases at WHO grade III) who did not receive any other treatment prior to surgery from The Third Affiliated Hospital of Nanchang University. The normal brain tissues (n = 10) were collected from healthy donors who died in traffic accidents. The written informed consents were obtained from every patient. This study was authorized via the Ethics Committee of our university.

## Cell culture, Sev exposure, and transfection

Glioma cell lines (LN229, T98 and A172) were provided via Procell (Wuhan, China), and grown in Dulbecco’s Modified Eagle’s Medium (DMEM) (Gibco, Grand Island, NY, USA) adding with 10% fetal bovine serum (Zhejiang Tianhang Biotechnology, Huzhou, China) and 1% antibiotics (Gibco) at 37°C in 5% CO_2_. Normal human astrocytes (NHA) were purchased from Cell applications (San Diego, CA, USA), and maintained in HA growth medium (Cell applications) containing growth supplement under 37°C and 5% CO_2_.

LN229 and T98 cells were exposed to various doses (1.7%, 3.4% and 5.1%) of Sev (Sigma-Aldrich, St. Louis, MO, USA) for 6 h via culturing in airtight glass chamber that was connected to an anesthetic vaporizer (DRE, Louisville, KY, USA), and Sev concentration was monitored using a gas monitor (Drager, Lubeck, Germany). Then cells were cultured in normal condition for 24 h prior to further experiments.

HMMR-AS1 or CDK4 overexpression vector was generated usingpcDNA3.1 vector (Thermo Fisher Scientific, Waltham, MA, USA), and pcDNA3.1 vector functioned as negative control. siRNA for HMMR-AS1 (si-HMMR-AS1), siRNA negative control (si-con), miR-7 mimic, negative control of mimic (miR-con), miR-7 inhibitor (in-miR-7), and negative control of inhibitor (in-miR-con) were formed via Ribobio (Guangzhou, China). The related sequences are displayed in Table 2. LN229 and T98 cells were transfected with above vectors or oligos using Lipofectamine 3000 (Thermo Fisher Scientific) for 24 h.

**Table 2.** The sequences for transfection in this study

Name	Sequence (5'-3')
si-HMMR-AS1	UUCUAGUGGCUUCUACUUGGC
si-con	AAGACAUUGUGUGUCCGCCTT
miR-7 mimic	UGGAAGACUAGUGAUUUUGUUGU
miR-con	CGAUCGCAUCAGCAUCGAUUGC
in-miR-7	ACAACAAAAUCACUAGUCUUCCA
in-miR-con	CUAACGCAUGCACAGUCGUACG

## Quantitative reverse transcription polymerase chain reaction (qRT-PCR)

RNA was isolated via Trizol (Beyotime, Shanghai, China) according to the protocols in a previous report [25], and reversely transcribed to complementary DNA (cDNA) with the specific reverse transcription kit (Thermo Fisher Scientific). The cDNA together with SYBR Green (Thermo Fisher Scientific) and primers was used for qRT-PCR. The specific primers were synthesized via Sangon (Shanghai, China) and displayed in Table 3. Glyceraldehyde-3-phosphate dehydrogenase (GAPDH) or U6 functioned as reference gene. The relative RNA level was calculated according to2^−ΔΔCt^ method [26].

**Table 3.** The primer sequences for qRT-PCR in this study

Name	Sequence (5'-3')	
	Forward	Reverse
miR- 6875-3p	GCCGAGATTCTTCCTGCCCTG	CAGTGCGTGTCGTGGAGT
miR-299-5p	GCCGAGTGGTTTACCGTCCCAC	CAGTGCGTGTCGTGGAGT
miR-627-3p	GCCGAGTCTTTTCTTTGAGAC	CAGTGCGTGTCGTGGAGT
miR-8087	GCCGAGGAAGACTTCTTGGA	CAGTGCGTGTCGTGGAGT
miR-3911	GCCGAGTGTGTGGATCCTGGA	CAGTGCGTGTCGTGGAGT
miR-7	GCCGAGTGGAAGACTAGTGA	CAGTGCGTGTCGTGGAGT
miR-18b	GCCGAGTAAGGTGCATCTAGT	CAGTGCGTGTCGTGGAGT
miR-6871	GCCGAGCATGGGAGTTCGGGG	CAGTGCGTGTCGTGGAGT
miR-5581	GCCGAGAGCCTTCCAGGAGAA	CAGTGCGTGTCGTGGAGT
miR-7151	GCCGAGGATCCATCTCTGCCT	CAGTGCGTGTCGTGGAGT
U6	GCTCGCTTCGGCAGCACA	GAGGTATTCGCACCAGAGGA
HMMR-AS1	GCATCCTTTGGTTTGAGAGAGA	AACTGTCCCTTGGCTTGCTT
CDK4	GTCTATGGTCGGGCCCTCT	CCATAGGCACCGACACCAAT
GAPDH	GAAAGCCTGCCGGTGACTAA	TTCCCGTTCTCAGCCTTGAC

## Western blot

The western blot assay was conducted as previously reported [27]. In brief, cells were lysed in radio-immunoprecipitation assay buffer (Beyotime), and protein concentration was determined via bicinchoninic acid assay kit (Thermo Fisher Scientific). 30 μg protein was separated via10% sodium dodecyl sulfate-polyacrylamide gel electrophoresis and transferred on polyvinylidene fluoride membranes (Bio-Rad, Hercules, CA, USA), which were blocked in 5% fat-free milk. The membranes were incubated with primary antibodies anti-CDK4 (ab137675, 1:3000 dilution, Abcam, Cambridge, UK), and anti-β-actin (ab115777, 1:500 dilution, Abcam) overnight, and IgG labeled via horseradish peroxidase (ab205718, 1:20,000 dilution, Abcam) for 2 h. Next, the membranes were exposed to BeyoECL Plus kit (Beyotime). β-actin functioned as reference. Relative protein level was analyzed according to the gray value of blot that was detected via Quantity One (Bio-Rad).

## Cell counting kit-8 (CCK-8)

CCK-8 method was used to investigate cell viability referring to a previous study [28]. After the transfection or Sev treatment, 1 × 10^4^ LN229 and T98 cells were placed into 96-well plates. After incubation for 48 h, 10 μL CCK-8 (Beyotime) was added. Next, cells were cultured for 3 h, and the optical density value at 450 nm was examined with a microplate reader (Bio-Rad). Cell viability was normalized to the control group× 100%.

## Colony formation analysis

Colony formation assay was conducted as previously reported [29]. After the transfection or Sev exposure, 1 × 10^3^ LN229 and T98 cells were inoculated into 6-well plates. Following incubation for 2 weeks, clones were fixed with 4% paraformaldehyde (Beyotime) and stained via 0.1% crystal violet (Solarbio, Beijing, China). The images of colony formation were photographed. The colony formation ratio was normalized to the control group× 100%.

## Transwell analysis

Transwell invasion assay was performed according to a previous report with some modifications [30]. Cell invasion was analyzed with 24-well transwell chambers (Corning, Corning, NY, USA) precoated via Matrigel (Solarbio). After the transfection or Sev exposure, 3 × 10^5^ LN229 and T98 cells in DMEM without serum were placed into upper chambers. The low chambers were added with 600 μL DMEM plus 10% serum. Following culture for 8 h, the invasive cells were stained by 0.1% crystal violet. The cells were imaged under 100 × magnification microscope (Olympus, Tokyo, Japan), and counted by Image J software (NIH, Bethesda, MD, USA).

## Dual-luciferase reporter and RNA pull-down analyses

The dual-luciferase reporter and RNA pull-down assays were performed according to a previous report [30]. The wild-type sequence of HMMR-AS1 or CDK4 3ʹ untranslated region (UTR) with miR-7 binding sequence was cloned into pGL3-Basic vector (Promega, Madison, WI, USA), generating HMMR-AS1-WT and CDK4-WT. The mutant-type luciferase reporter vectors HMMR-AS1-MUT and CDK4-MUT were formed via mutating the binding sites of miR-7. LN229 and T98 cells were transfected with these luciferase reporter vectors and miR-7 mimic or miR-con. The luciferase activity was detected via a dual-luciferase assay kit (Promega) after 24 of post-transfection.

RNA pull-down analysis was performed with Magnetic RNA-Protein Pull-Down kit (Thermo Fisher Scientific). The biotin labeled Bio-HMMR-AS1 probe and Bio-miR-7 were constructed via Viagene (Changzhou, China).1 × 10^7^ LN229 and T98 cells were lysed and incubated with streptavidin magnetic beads overnight. RNA on the beads was isolated, and HMMR-AS1, the relative miRNAs and CDK4 mRNA levels were examined by qRT-PCR.

## Xenograft experiment

The xenograft experiments were performed according to a previous report [31]. Four-week-old male BALB/c nude mice were purchased from Vital River (Beijing, China). LN229 cells stably transfected with HMMR-AS1 overexpression vector or pcDNA were exposed to 5.1% Sev or not, and then subcutaneously injected into mice. The mice were divided into control, Sev + pcDNA, or Sev + HMMR-AS1 group (n = 6). Tumor size was examined weekly, and calculated according to volume = 0.5 × length × width^2^. After 4 weeks, mice were euthanized using 5% isoflurane (Sigma, St. Louis, MO, USA). The tumors were dissected out and weighed, and then collected for measurement of HMMR-AS1, miR-7 and CDK4 levels. The animal experiments were permitted via the Animal Ethics Committee of The Third Affiliated Hospital of Nanchang University.

## Statistical analysis

The data from three independent experiments were displayed as mean ± standard deviation (SD). The linear correlation of gene level was tested by Pearson correlation analysis. The statistical analysis was conducted using GraphPad Prism 8 (GraphPad, La Jolla, CA, USA). The difference for two groups was detected by Student’s *t*-test, and that for multiple groups was analyzed via analysis of variance (ANOVA) followed via Tukey post hoc test as appropriate. *P* < 0.05 indicated the significance.

## Results

### HMMR-AS1 abundance is enhanced in glioma

**Figure 1.**
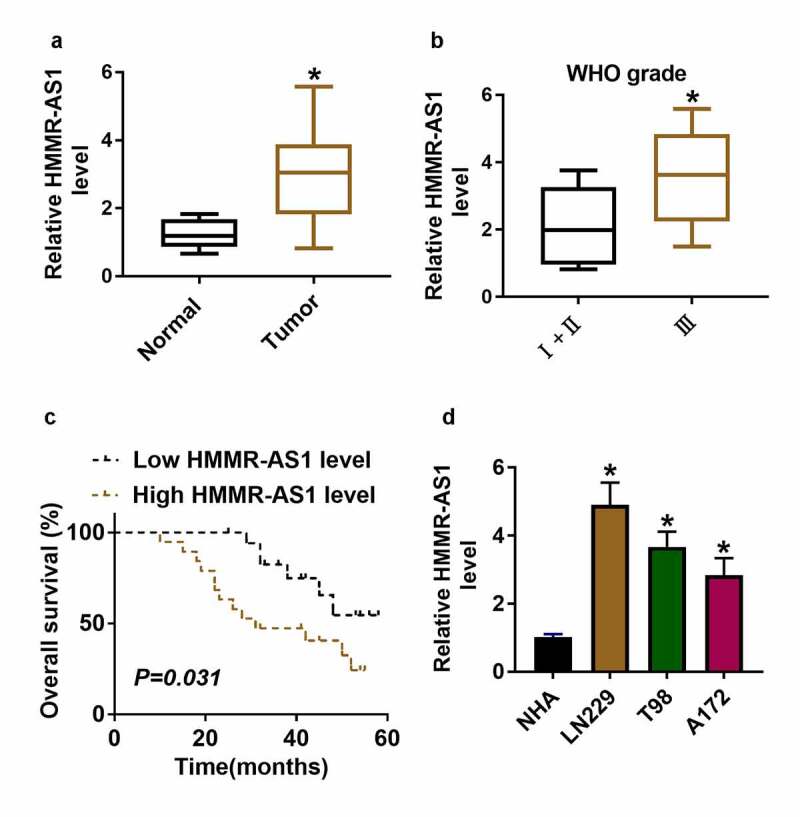
HMMR-AS1 expression in glioma. (a) HMMR-AS1 level was measured in glioma tissues (n = 37) and normal samples (n = 10). (b) HMMR-AS1 level in glioma patients at different grades. (c) The association between HMMR-AS1 level and overall survival in glioma patients. (d) HMMR-AS1 level was measured in glioma cells (LN229, T98 and A172) and normal human astrocytes (NHA). *P < 0.05

Sev has a suppressive role in glioma development. The purpose of this work is to explore what role Sev plays and how it works in glioma. It was hypothesized that Sev could target the HMMR-AS1/miR-7/CDK4 axis to inhibit glioma progression. To explore whether HMMR-AS1 was related to glioma progression, HMMR-AS1 expression change was examined in glioma. HMMR-AS1 abundance was significantly higher in glioma tissues and serum samples (n = 37) than in normal samples (n = 10) (Figure 1a**, supplementary Figure S1**). And HMMR-AS1 level was increased in patients at advanced stage (WHO grade III; n = 17) compared with those at early stage (WHO grades I and II; n = 20) (Figure 1b). Furthermore, patients were divided into low (n = 18) and high (n = 19) HMMR-AS1 level group according to the median value of HMMR-AS1 level in glioma patients. Patients with high HMMR-AS1 level had lower overall survival (*P* = 0.031) (Figure 1c). Moreover, HMMR-AS1 abundance was measured in glioma cell lines (LN229, T98 and A172) and control NHA cells. Results showed that HMMR-AS1 abundance was increased more than 2.8-fold in glioma cells compared with NHA cells (Figure 1d). Taken together, increased HMMR-AS1 might be associated with glioma progression. LN229 and T98 cells with highest expression of HMMR-AS1 were selected for further experiments.

## Sev inhibits cell viability, invasion, and colony formation by regulating HMMR-AS1 in glioma cells

**Figure 2.**
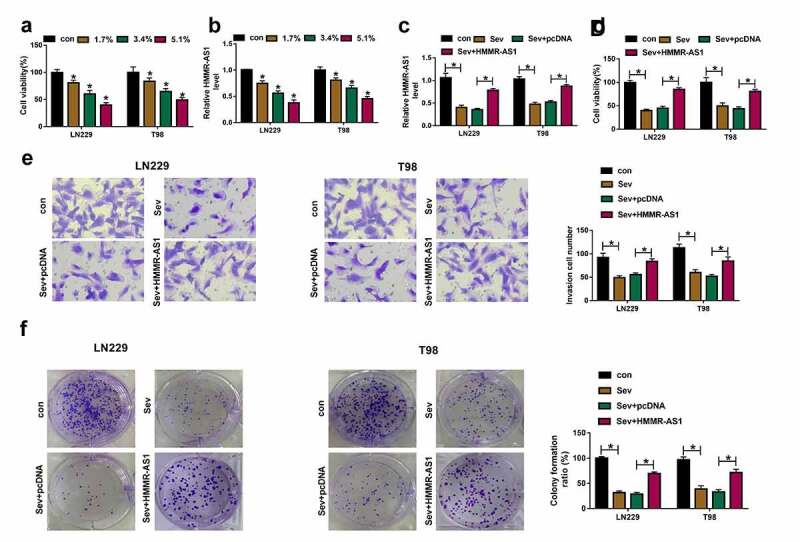
The influence of Sev and HMMR-AS1 on glioma progression. (a) Cell viability was detected in LN229 and T98 cells after stimulation of different doses of Sev. (b) HMMR-AS1 abundance was examined in LN229 and T98 cells after stimulation of various doses of Sev. (c) HMMR-AS1 level was detected in LN229 and T98 cells with transfection of HMMR-AS1 overexpression vector or pcDNA after treatment of 5.1% Sev. Cell viability (d), invasion (e), colony formation (f) were examined in LN229 and T98 cells transfected with HMMR-AS1 overexpression vector or pcDNA after treatment of 5.1% Sev. *P < 0.05

To analyze whether HMMR-AS1 was associated with the function of Sev in glioma, the influence of Sev on glioma cell viability and HMMR-AS1 expression was assessed. Exposure to Sev significantly decreased viability of LN229 and T98 cells and HMMR-AS1 expression in a dose-dependent pattern (Figure 2(a,B)). The 5.1% Sev was used for further experiments. Furthermore, HMMR-AS1 abundance was up-regulated via transfection of HMMR-AS1 overexpression vector in the two cell lines under Sev treatment (Figure 2c). Additionally, HMMR-AS1 up-regulation attenuated Sev-induced viability inhibition in LN229 and T98 cells (Figure 2d). Moreover, Sev evidently suppressed invasion of LN229 and T98 cells, which was mitigated via HMMR-AS1 overexpression (Figure 2e). In addition, Sev significantly restrained colony formation ability of the two cell lines, and this effect was relieved by HMMR-AS1 restoration (figure 2f). These results suggested that Sev suppressed glioma progression by decreasing HMMR-AS1.

## HMMR-AS1 targets miR-7 in glioma cells

**Figure 3.**
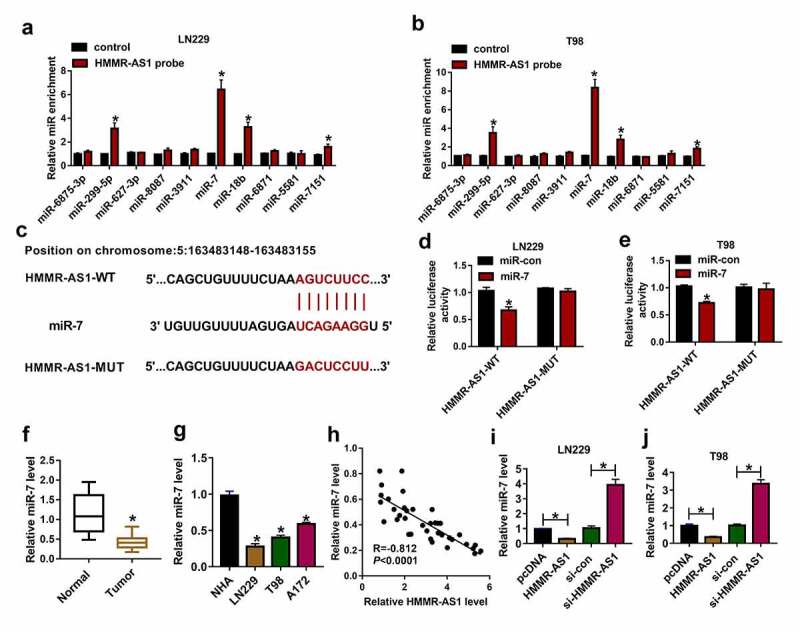
The target association of HMMR-AS1 and miR-7 in glioma cells. (a and b) The enrichment levels of top 10 miRNAs were measured in LN229 and T98 cells after RNA pull-down using HMMR-AS1 probe. (c) The binding sequence of HMMR-AS1 and miR-7 was predicted via LncBase V.2. (d and e) Luciferase activity was examined in LN229 and T98 cells transfected with HMMR-AS1-WT or HMMR-AS1-MUT and miR-7 mimic or miR-con. (f and g) miR-7 level was examined in glioma tissues and cells. (h) The linear correlation of HMMR-AS1 and miR-7 in glioma tissues. (i and j) miR-7 expression was examined in LN229 and T98 cells with transfection of pcDNA, HMMR-AS1 overexpression vector, si-con or si-HMMR-AS1. *P < 0.05

To analyze the regulatory network of HMMR-AS1 in glioma, the targeted miRNAs of HMMR-AS1 were predicted via LncBase V.2, and the top 10 predicted miRNAs were selected. These miRNAs were tested via RNA pull-down using HMMR-AS1 probe. This probe effectively enriched higher level of miR-299-5p, miR-7, miR-18b and miR-7151 (Figure 3(a,B)). miR-7 with highest enrichment level was selected for further experiments. The complementary sequence of HMMR-AS1 and miR-7 was displayed in Figure 3c. HMMR-AS1-WT and HMMR-AS1-MUT were constructed. miR-7 overexpression induced more than 60% loss of luciferase activity in the HMMR-AS1-WT group, while it did not alter the luciferase activity in the HMMR-AS1-MUT group (Figure 3(d,E)). Moreover, low expression of miR-7 was measured in glioma tissues and cells (Figure 3(f,G)). Meanwhile, miR-7 level was negatively correlated with HMMR-AS1 expression in glioma tissues (Figure 3h). Furthermore, miR-7 abundance was negatively regulated via HMMR-AS1 in LN229 and T98 cells (Figure 3(i,J)). These data indicated that miR-7 was targeted via HMMR-AS1 in glioma cells.

## HMMR-AS1 silence constrains cell viability, invasion, and colony formation by increasing miR-7 in glioma cells

**Figure 4.**
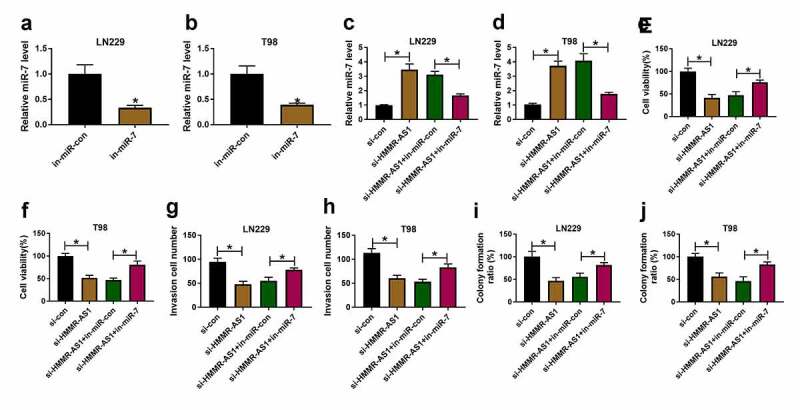
The effect of HMMR-AS1 and miR-7 on glioma progression. (a and b) miR-7 level was detected in LN229 and T98 cells transfected with in-miR-con or in-miR-7. miR-7 level (c and d), viability (e and f), invasion (g and h), colony formation ration (i and j) were determined in LN229 and T98 cells transfected with si-con, si-HMMR-AS1, si-HMMR-AS1 + in-miR-con or in-miR-7. *P < 0.05

To explore the role of HMMR-AS1/miR-7 axis in glioma progression, LN229 and T98 cells were transfected with si-con, si-HMMR-AS1, si-HMMR-AS1 + in-miR-con or in-miR-7. Transfection with in-miR-7 effectively decreased miR-7 expression in LN229 and T98 cells compared with in-miR-con group (Figure 4(a,B)). Moreover, miR-7 abundance was evidently increased via HMMR-AS1 silence in the two cell lines, which was reduced via introduction of in-miR-7 (Figure 4(c,D)). Additionally, cell viability was markedly decreased by HMMR-AS1 silence, and transfection with in-miR-7 mitigated this effect (Figure 4(e,F)). Furthermore, HMMR-AS1 silence significantly constrained invasion of LN229 and T98 cells, which was mitigated via miR-7 knockdown (Figure 4(g,H)). In addition, HMMR-AS1 interference markedly repressed colony formation ability of LN229 and T98 cells, and this influence was alleviated by miR-7 down-regulation (Figure 4(i,J)). These results showed that HMMR-AS1 knockdown repressed glioma progression via regulating miR-7.

## Sev up-regulates miR-7 expression via decreasing HMMR-AS1 in glioma cells

**Figure 5.**
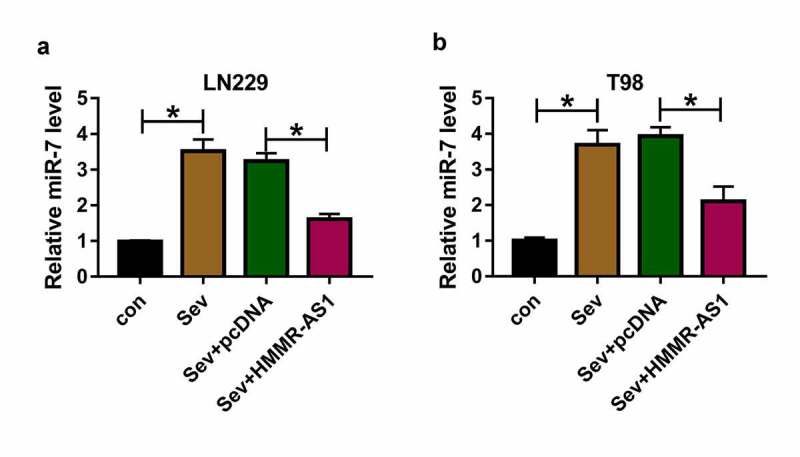
The effect of Sev and HMMR-AS1 on miR-7 expression in glioma cells. (a and b) miR-7 level was measured in LN229 and T98 cells transfected with pcDNA or HMMR-AS1 overexpression vector after treatment of 5.1% Sev. *P < 0.05

To study whether Sev could regulate miR-7 expression via HMMR-AS1, miR-7 expression was detected in LN229 and T98 cells with overexpression of HMMR-AS1 under Sev treatment. As exhibited in Figure 5(a,B), miR-7 abundance was significantly increased by Sev treatment, and this effect was mitigated via HMMR-AS1 overexpression. These data indicated Sev could increase miR-7 level by regulating HMMR-AS1.

## CDK4 is targeted by miR-7 in glioma cells

**Figure 6.**
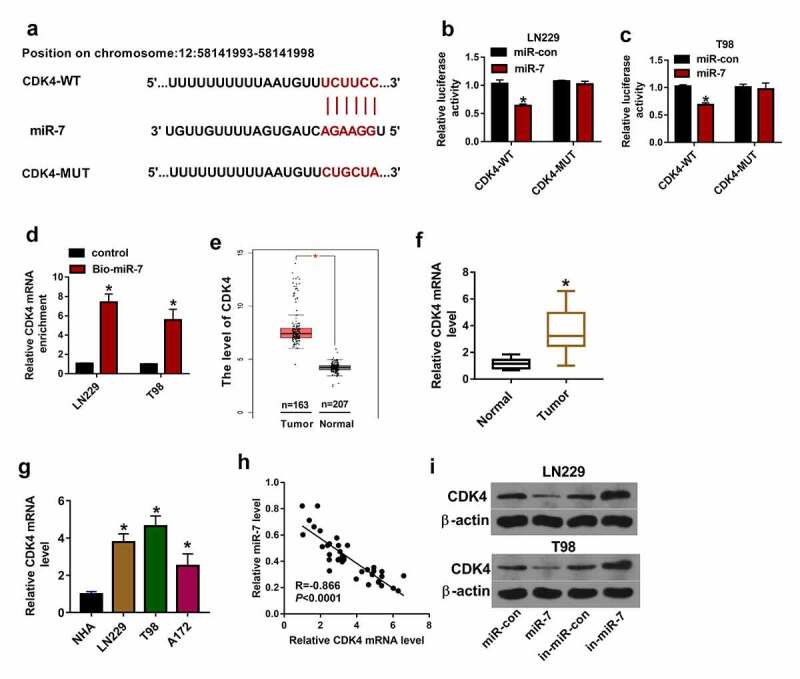
The target relationship of miR-7 and CDK4 in glioma cells. (a) The binding sequence of miR-7 and CDK4 was predicted via microT-CDS. (b and c) Luciferase activity was detected in LN229 and T98 cells transfected with CDK4-WT or CDK4-MUT and miR-7 mimic or miR-con. (d) miR-7 and CDK4 mRNA levels were measured after RNA pull-down using Bio-miR-7. (e) CDK4 expression in glioma tissues was analyzed via GEPIA. (f and g) CDK4 mRNA level was detected in glioma tissues and cells. (h) The linear correlation of miR-7 and CDK4 in glioma tissues. (i and j) CDK4 protein level was examined in LN229 and T98 cells transfected with miR-con, miR-7 mimic, in-miR-con or in-miR-7. *P < 0.05

To further underlie the regulatory network involved in HMMR-AS1/miR-7 axis in glioma, the potential downstream genes were searched. The top 10 up-regulated genes in glioma tissues were predicted via GEPIA, and the related expression information is shown in Table 1. CDK4 was the mostly increased gene in glioma. microT-CDS analyzed and displayed there was binding site between miR-7 and CDK4 (Figure 6a). To validate their interaction, dual-luciferase reporter and RNA pull-down analyses were performed. miR-7 up-regulation led to clear reduction of luciferase activity in the CDK4-WT group, but it did not change the luciferase activity in the CDK4-MUT group (Figure 6(b,C)). Furthermore, RNA pull-down analysis exhibited miR-7 effectively enriched CDK4 in LN229 and T98 cells (Figure 6d). High expression of CDK4 in glioma tissues was predicted via GEPIA (Figure 6e). Moreover, CDK4 mRNA level was detected in glioma tissues and cells, and results showed that CDK4 abundance was significantly increased (Figure 6(f,G)). miR-7 abundance in glioma tissues was negatively associated with CDK4 mRNA level (Figure 6h). Additionally, CDK4 protein expression was negatively regulated by miR-7 in LN229 and T98 cells (Figure 6i). These results indicated miR-7 could target CDK4 in glioma cells.

## miR-7 overexpression restrains cell viability, invasion, and colony formation via reducing CDK4 in glioma cells

**Figure 7.**
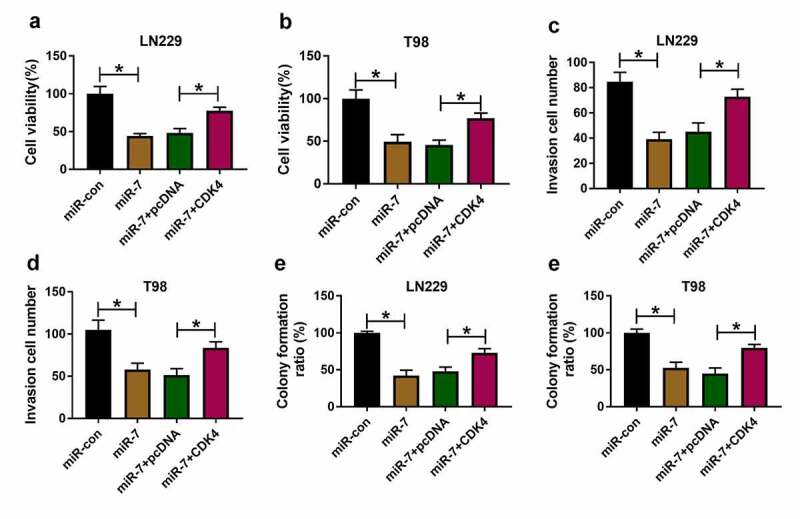
The effect of miR-7 and CDK4 on glioma progression. Cell viability (a and b), invasion (c and d), and colony formation ration (e and f) were determined in LN229 and T98 cells transfected with miR-con, miR-7 mimic, miR-7 mimic + pcDNA or CDK4 overexpression vector. *P < 0.05

To test the function of miR-7/CDK4 axis in glioma progression, LN229 and T98 cells were transfected with miR-con, miR-7 mimic, miR-7 mimic + pcDNA or CDK4 overexpression vector. miR-7 overexpression obviously decreased cell viability, and CDK4 up-regulation mitigated this effect (Figure 7(a,B)). Moreover, miR-7 overexpression evidently inhibited invasion of the two cell lines, and this effect was abolished by CDK4 up-regulation (Figure 7(c,D)). Additionally, miR-7 overexpression significantly constrained colony formation ability of LN229 and T98 cells, which was attenuated via CDK4 addition (Figure 7(e,F)). These data showed miR-7 overexpression constrained glioma progression by targeting CDK4.

## Sev reduces CDK4 expression by regulating HMMR-AS1/miR-7 axis in glioma cells

**Figure 8.**
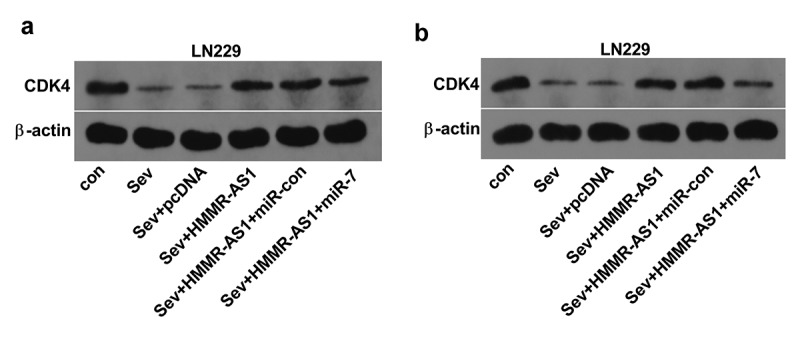
The effect of Sev, HMMR-AS1 and miR-7 on CDK4 expression in glioma cells. (a) CDK4 protein level was examined in LN229 and T98 cells transfected with pcDNA, HMMR-AS1 overexpression vector, HMMR-AS1 overexpression vector + miR-con or miR-7 mimic after treatment of 5.1% Sev. *P < 0.05

To analyze whether Sev could modulate CDK4 abundance by HMMR-AS1/miR-7 axis, CDK4 protein level was measured in LN229 and T98 cells with indicated transfection under Sev exposure. As exhibited in Figure 8(a,B), CDK4 protein abundance was obviously decreased via Sev exposure. Moreover, HMMR-AS1 overexpression attenuated Sev-mediated reduction of CDK4 level, and this effect was abrogated via miR-7 overexpression. These results suggested Sev could decrease CDK4 expression via HMMR-AS1/miR-7 axis.

## Sev reduces tumor growth by decreasing HMMR-AS1 in glioma

**Figure 9.**
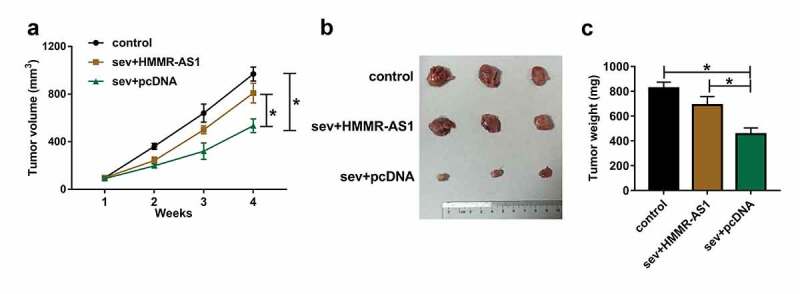
The effect of Sev and HMMR-AS1 on tumor growth. LN229 cells stably transfected with HMMR-AS1 overexpression vector or pcDNA were exposed to 5.1% Sev or not and then injected into the mice to generate xenograft tumor. (a) Tumor volume was examined weekly. (b) The tumor images in each group. (c) Tumor weight was detected in each group. n = 6. *P < 0.05

To explore the anti-tumor role of Sev in glioma *in vivo*, we performed the xenograft experiments using LN229 cells stably transfected with HMMR-AS1 overexpression vector or pcDNA after exposure to 5.1% Sev or not. Tumor volume and weight were obviously decreased in Sev + pcDNA group compared with control group, which were reversed in Sev + HMMR-AS1 group (Figure 9(a-C)). These results suggested Sev inhibited glioma cell growth in vivo by regulation of HMMR-AS1.

## Discussion

Glioma accounts for ~30% of brain tumors with high mortality [32]. This tumor has proved challenging to cure [33]. The exposure to anesthetic gases like Sev opens promising option for glioma treatment by inhibiting glioma progression [2]. Exploring the mechanism allows Sev in glioma treatment helps understand the pharmacological effect of Sev. This study is the first to identify that Sev can play the anti-glioma role via regulating HMMR-AS1/miR-7/CDK4 axis.

Multiple studies suggested Sev could inhibit glioma cell migration and invasion by interacting with miRNAs, like miR-34a-5p, miR-146b-5p and miR-637 [4,34,35]. Additionally, Sev could repress cell proliferation and increase apoptosis of glioma cells [5,31,36]. These reports indicated the anti-glioma role of Sev by reducing cell proliferation, migration and invasion and increasing apoptosis. Similarly, we also found this function of Sev in glioma cells. However, it was opposite to the study of Lai *et al*., which showed exposure to 4% Sev for 4 h contributed to cell migration, invasion and colony formation in glioma by regulating cell surface protein 44 [37]. We hypothesized that changed doses and exposure times of Sev might induce different response in glioma, and lower dose and shorter exposure time of Sev might not exhibit the anti-tumor role. Our study indicated the anti-glioma function of Sev (5.1% for 6 h) *in vitro*.

LncRNAs are involved in the pathology of brain tumors, including glioma [38]. Moreover, lncRNA-mediated networks are responsible for understanding the activity of Sev [10], such as lncRNA metastasis-associated lung adenocarcinoma transcript 1 and small nucleolar RNA host gene 1 [39,40]. Here we were the first to confirm HMMR-AS1 was decreased via Sev in glioma cells. Previous studies suggested that HMMR-AS1 played as an oncogenic lncRNA in glioma [14], and other tumors [11–13]. This work also found the anti-tumor function of HMMR-AS1 knockdown in glioma, and Sev could restrain glioma progression via decreasing HMMR-AS1.

Next, we wanted to explore a lncRNA/miRNA/mRNA network mediated via HMMR-AS1 in glioma. Many brain miRNAs are dysregulated in response to Sev exposure [41]. Here we confirmed miR-7 was targeted by HMMR-AS1 and lowly expressed in glioma. Li *et al*. reported that miR-7 could repress proliferation, migration and invasion and trigger apoptosis of glioma cells through regulating neuro-oncological ventral antigen 2 [42]. Bhere *et al*. showed that miR-7 could enhance glioma cell apoptosis [43]. Moreover, miR-7 suppressed tumor angiogenesis and growth of glioma in a murine xenograft model [44]. Additionally, miR-7 is reported to inhibit growth of glioma cells and xenograft models by blocking the phosphoinositide-3-kinase (PI3K)/protein kinase B (ATK) and Raf/mitogen-activated protein kinase (MEK)/extracellular signal-regulated kinase (ERK) pathways [45]. These reports suggested the anti-tumor function of miR-7 in glioma. In agreement with these reports, we also validated miR-7 inhibited glioma progression. Moreover, we found that Sev could regulate miR-7 via HMMR-AS1 to participate in glioma development.

We next identified CDK4 was targeted via miR-7 in glioma. Previous studies reported CDK4 could facilitate glioma cell proliferation and restrain apoptosis [21,46]. More importantly, CDK4 was reported to be regulated via Sev in glioma [5]. Similarly, we also found the oncogenic role of CDK4 in glioma by promoting colony formation. Moreover, multiple reports indicated that CDK4 could promote cell invasion in human tumors, such as non-small-cell lung cancer, gastric cancer and colon cancer [47–49]. However, there is no report in support of the role of CDK4 in glioma cell invasion. This study found that CDK4 might promote glioma cell invasion. Furthermore, we found that Sev could reduce CDK4 expression via regulating HMMR-AS1/miR-7 axis. In this way, Sev attenuated glioma progression *in vitro*. Additionally, we established the xenograft model, and further confirmed the anti-tumor role of Sev.

However, there are some limitations in this study. Previous reports indicated the serum lncRNAs might function as diagnostic and prognostic biomarkers for glioma patients [50,51]. Here we also found HMMR-AS1 expression was increased in serum of glioma patients, indicating the potential of HMMR-AS1 in diagnosis and prognosis of glioma patients. Hence, the clinical role of HMMR-AS1 would be explored in future. Moreover, only 37 patients were obtained in the current study. Hence, we need more samples to better investigate the clinical value of HMMR-AS1 to obtain more reliable conclusions. Additionally, microRNA, fungi and algae may have important roles in fighting cancer and COVID-19 [52–54]. Whether they could prevent glioma development remains further study in future.

## Conclusion

Sev exhibits an anti-tumor role in glioma, possibly via regulating HMMR-AS1/miR-7/CDK4 axis. This study proposes a novel insight into the pharmacological function of Sev in glioma.

## Supplementary Material

Supplemental Material

## Data Availability

The data and material presented in this manuscript is available from the corresponding author on reasonable request.

## Highlights

1. HMMR-AS1 level is increased in glioma tissues and cells.

2. HMMR-AS1 expression is decreased via Sevoflurane in glioma cells.

3. Sevoflurane restrains glioma progression by decreasing HMMR-AS1.

4. Sevoflurane regulates the HMMR-AS1/miR-7/CDK4 axis in glioma cells.

## References

[1] Kristensen BW, Priesterbach-Ackley LP, Petersen JK, et al. Molecular pathology of tumors of the central nervous system. Ann Oncol. 2019;30(8):1265–1278. 31124566 10.1093/annonc/mdz164 PMC6683853

[2] Chen X, Mao YG, Yu ZQ, et al. Potential rules of anesthetic gases on glioma. Med Gas Res. 2020;10(1):50–53. 32189670 10.4103/2045-9912.279984 PMC7871937

[3] Brohan J, Goudra BG. The Role of GABA Receptor Agonists in Anesthesia and Sedation. CNS Drugs. 2017;31(10):845–856. 29039138 10.1007/s40263-017-0463-7

[4] Zhao H, Xing F, Yuan J, et al. Sevoflurane inhibits migration and invasion of glioma cells via regulating miR-34a-5p/MMP-2 axis. Life Sci. 2020;256:117897. 32502543 10.1016/j.lfs.2020.117897

[5] Xu W, Xue R, Xia R, et al. Sevoflurane impedes the progression of glioma through modulating the circular RNA has_circ_0012129/miR-761/TGIF2 axis. Eur Rev Med Pharmacol Sci. 2020;24:5534–5548. 32495888 10.26355/eurrev_202005_21339

[6] Gao C, Shen J, Meng ZX, et al. Sevoflurane inhibits glioma cells proliferation and metastasis through miRNA-124-3p/ROCK1 Axis. Pathol Oncol Res. 2020;26(2):947–954. 30915607 10.1007/s12253-019-00597-1

[7] Rynkeviciene R, Simiene J, Strainiene E, et al. Non-Coding RNAs in Glioma. Cancers (Basel). 2018;11(1):17. 10.3390/cancers11010017 PMC6356972 30583549

[8] Tao C, Luo H, Chen L, et al. Identification of an epithelial-mesenchymal transition related long non-coding RNA (LncRNA) signature in Glioma. Bioengineered. 2021;12(1):4016–4031. 34288803 10.1080/21655979.2021.1951927 PMC8806607

[9] Zhang Y, Zhang Y, Wang S, et al. SP1-induced lncRNA ZFPM2 antisense RNA 1 (ZFPM2-AS1) aggravates glioma progression via the miR-515-5p/Superoxide dismutase 2 (SOD2) axis. Bioengineered. 2021;12(1):2299–2310. 34077295 10.1080/21655979.2021.1934241 PMC8806534

[10] Chen X, Zhou X, Lu D, et al. Aberrantly expressed long noncoding RNAs are involved in sevoflurane-induced developing hippocampal neuronal apoptosis: a microarray related study. Metab Brain Dis. 2016;31(5):1031–1040. 27234990 10.1007/s11011-016-9838-6

[11] Liu W, Ma J, Cheng Y, et al. HMMR antisense RNA 1, a novel long noncoding RNA, regulates the progression of basal-like breast cancer cells. Breast Cancer (Dove Med Press). 2016;8:223–229. 27920576 10.2147/BCTT.S119997 PMC5125767

[12] Chu ZP, Dai J, Jia LG, et al. Increased expression of long noncoding RNA HMMR-AS1 in epithelial ovarian cancer: an independent prognostic factor. Eur Rev Med Pharmacol Sci. 2018;22:8145–8150. 30556852 10.26355/eurrev_201812_16506

[13] Cai Y, Sheng Z, Chen Y, et al. LncRNA HMMR-AS1 promotes proliferation and metastasis of lung adenocarcinoma by regulating MiR-138/sirt6 axis. Aging (Albany NY). 2019;11:3041–3054. 31128573 10.18632/aging.101958 PMC6555459

[14] Li J, Ji X, Wang H. Targeting long noncoding RNA HMMR-AS1 suppresses and radiosensitizes glioblastoma. Neoplasia. 2018;20(5):456–466. 29574252 10.1016/j.neo.2018.02.010 PMC5915996

[15] Anthiya S, Griveau A, Loussouarn C, et al. MicroRNA-based drugs for brain tumors. Trends Cancer. 2018;4(3):222–238. 29506672 10.1016/j.trecan.2017.12.008

[16] Zhou Q, Liu J, Quan J, et al. MicroRNAs as potential biomarkers for the diagnosis of glioma: a systematic review and meta-analysis. Cancer Sci. 2018;109(9):2651–2659. 29949235 10.1111/cas.13714 PMC6125451

[17] Wang B, Sun F, Dong N, et al. MicroRNA-7 directly targets insulin-like growth factor 1 receptor to inhibit cellular growth and glucose metabolism in gliomas. Diagn Pathol. 2014;9(1):211. 25394492 10.1186/s13000-014-0211-y PMC4236426

[18] Shukla A, Gupta P, Singh R, et al. Glycolytic inhibitor 2-Deoxy-d-Glucose activates migration and invasion in glioblastoma cells through modulation of the miR-7-5p/TFF3 signaling pathway. Biochem Biophys Res Commun. 2018;499(4):829–835. 29621542 10.1016/j.bbrc.2018.04.001

[19] Chou J, Quigley DA, Robinson TM, et al. Transcription-associated cyclin-dependent kinases as targets and biomarkers for cancer therapy. Cancer Discov. 2020;10(3):351–370. 32071145 10.1158/2159-8290.CD-19-0528

[20] Du Q, Guo X, Wang M, et al. The application and prospect of CDK4/6 inhibitors in malignant solid tumors. J Hematol Oncol. 2020;13(1):41. 32357912 10.1186/s13045-020-00880-8 PMC7195725

[21] Moradimotlagh A, Arefian E, Rezazadeh Valojerdi R, et al. MicroRNA-129 inhibits glioma cell growth by targeting CDK4, CDK6, and MDM2. Mol Ther Nucleic Acids. 2020;19:759–764. 31954330 10.1016/j.omtn.2019.11.033 PMC6965505

[22] Tang Z, Li C, Kang B, et al. GEPIA: a web server for cancer and normal gene expression profiling and interactive analyses. Nucleic Acids Res. 2017;45(W1):98–102. 10.1093/nar/gkx247 PMC5570223 28407145

[23] Paraskevopoulou MD, Vlachos IS, Karagkouni D, et al. DIANA-LncBase v2: indexing microRNA targets on non-coding transcripts. Nucleic Acids Res. 2016;44(D1):231–238. 10.1093/nar/gkv1270 PMC4702897 26612864

[24] Paraskevopoulou MD, Georgakilas G, Kostoulas N, et al. DIANA-microT web server v5.0: service integration into miRNA functional analysis workflows. Nucleic Acids Res. 2013;41(W1):169–173. 10.1093/nar/gkt393 PMC3692048 23680784

[25] Puch-Hau C, Sanchez-Tapia IA, Patino-Suarez V, et al. Evaluation of two independent protocols for the extraction of DNA and RNA from different tissues of sea cucumber Isostichopus badionotus. MethodsX. 2019;6:1627–1634. 31367529 10.1016/j.mex.2019.07.010 PMC6652131

[26] Livak KJ, Schmittgen TD. Analysis of relative gene expression data using real-time quantitative PCR and the 2(-Delta Delta C(T)) Method. Methods. 2001;25(4):402–408. 11846609 10.1006/meth.2001.1262

[27] Liu Z, Liu Q, Chen S, et al. Circular RNA Circ_0005564 promotes osteogenic differentiation of bone marrow mesenchymal cells in osteoporosis. Bioengineered. 2021;12(1):4911–4923. 34374320 10.1080/21655979.2021.1959865 PMC8806437

[28] Sun Y, Hou Z, Luo B, et al. Circular RNA circRNA_0082835 promotes progression and lymphatic metastasis of primary melanoma by sponging microRNA miRNA-429. Bioengineered. 2021;12(1):4159–4173. 34288815 10.1080/21655979.2021.1953822 PMC8806410

[29] Zhou D, Lin X, Wang P, et al. Circular RNA circ_0001162 promotes cell proliferation and invasion of glioma via the miR-936/ERBB4 axis. Bioengineered. 2021;12(1):2106–2118. 34057019 10.1080/21655979.2021.1932221 PMC8806513

[30] Wang J, Zhu W, Tao G, et al. Circular RNA circ-LRP6 facilitates Myc-driven tumorigenesis in esophageal squamous cell cancer. Bioengineered. 2020;11(1):932–938. 32867570 10.1080/21655979.2020.1809922 PMC8291805

[31] Li H, Xia T, Guan Y, et al. Sevoflurane regulates glioma progression by Circ_0002755/miR-628-5p/MAGT1 axis. Cancer Manag Res. 2020;12:5085–5098. 32669871 10.2147/CMAR.S242135 PMC7335772

[32] Weller M, Wick W, Aldape K, et al. Glioma. Nat Rev Dis Primers. 2015;1(1):15017. 27188790 10.1038/nrdp.2015.17

[33] Aldape K, Brindle KM, Chesler L, et al. Challenges to curing primary brain tumours. Nat Rev Clin Oncol. 2019;16(8):509–520. 30733593 10.1038/s41571-019-0177-5 PMC6650350

[34] Zhang L, Wang J, Fu Z, et al. Sevoflurane suppresses migration and invasion of glioma cells by regulating miR-146b-5p and MMP16. Artif Cells Nanomed Biotechnol. 2019;47(1):3306–3314. 31385537 10.1080/21691401.2019.1648282

[35] Yi W, Li D, Guo Y, et al. Sevoflurane inhibits the migration and invasion of glioma cells by upregulating microRNA-637. Int J Mol Med. 2016;38(6):1857–1863. 27840895 10.3892/ijmm.2016.2797

[36] Gao C, He XF, Xu QR, et al. Sevoflurane downregulates insulin-like growth factor-1 to inhibit cell proliferation, invasion and trigger apoptosis in glioma through the PI3K/AKT signaling pathway. Anticancer Drugs. 2019;30(7):e0744. 31305291 10.1097/CAD.0000000000000744

[37] Lai RC, Shan WR, Zhou D, et al. Sevoflurane promotes migration, invasion, and colony-forming ability of human glioblastoma cells possibly via increasing the expression of cell surface protein 44. Acta Pharmacol Sin. 2019;40(11):1424–1435. 30967592 10.1038/s41401-019-0221-0 PMC6889382

[38] Pop S, Enciu AM, Necula LG, et al. Long non-coding RNAs in brain tumours: focus on recent epigenetic findings in glioma. J Cell Mol Med. 2018;22(10):4597–4610. 30117678 10.1111/jcmm.13781 PMC6156469

[39] Hu X, Hu X, Huang G. LncRNA MALAT1 is involved in sevoflurane-induced neurotoxicity in developing rats. J Cell Biochem. 2019;120(10):18209–18218. 31190336 10.1002/jcb.29127

[40] Zhang N, Wang D, Yang X, et al. Long noncoding RNA small nucleolar RNA host gene 1 contributes to sevoflurane-induced neurotoxicity through negatively modulating microRNA-181b. Neuroreport. 2020;31(5):416–424. 32150149 10.1097/WNR.0000000000001430

[41] Lu Y, Jian MY, Ouyang YB, et al. Changes in rat brain MicroRNA expression profiles following sevoflurane and propofol anesthesia. Chin Med J (Engl). 2015;128(11):1510–1515. 26021509 10.4103/0366-6999.157676 PMC4733764

[42] Li G, Huang M, Cai Y, et al. Circ-U2AF1 promotes human glioma via derepressing neuro-oncological ventral antigen 2 by sponging hsa-miR-7-5p. J Cell Physiol. 2019;234(6):9144–9155. 30341906 10.1002/jcp.27591

[43] Bhere D, Tamura K, Wakimoto H, et al. microRNA-7 upregulates death receptor 5 and primes resistant brain tumors to caspase-mediated apoptosis. Neuro Oncol. 2018;20(2):215–224. 29016934 10.1093/neuonc/nox138 PMC5777493

[44] Babae N, Bourajjaj M, Liu Y, et al. Systemic miRNA-7 delivery inhibits tumor angiogenesis and growth in murine xenograft glioblastoma. Oncotarget. 2014;5(16):6687–6700. 25149532 10.18632/oncotarget.2235 PMC4196156

[45] Liu Z, Jiang Z, Huang J, et al. miR-7 inhibits glioblastoma growth by simultaneously interfering with the PI3K/ATK and Raf/MEK/ERK pathways. Int J Oncol. 2014;44:1571–1580. 24603851 10.3892/ijo.2014.2322

[46] Song D, Liang H, Qu B, et al. Ivermectin inhibits the growth of glioma cells by inducing cell cycle arrest and apoptosis in vitro and in vivo. J Cell Biochem. 2019;120(1):622–633. 30596403 10.1002/jcb.27420

[47] Wu B, Liu R. PAQR4 promotes cell proliferation and metastasis through the CDK4-pRB-E2F1 pathway in non-small-cell lung cancer. Onco Targets Ther. 2019;12:3625–3633. 31190865 10.2147/OTT.S181432 PMC6521844

[48] Fan HN, Zhu MY, Peng SQ, et al. Dihydroartemisinin inhibits the growth and invasion of gastric cancer cells by regulating cyclin D1-CDK4-Rb signaling. Pathol Res Pract. 2020;216(2):152795. 31879047 10.1016/j.prp.2019.152795

[49] Sun W, Nie W, Wang Z, et al. Lnc HAGLR promotes colon cancer progression through sponging miR-185-5p and activating CDK4 and CDK6 in vitro and in vivo. Onco Targets Ther. 2020;13:5913–5925. 32606801 10.2147/OTT.S246092 PMC7319508

[50] Tan SK, Pastori C, Penas C, et al. Serum long noncoding RNA HOTAIR as a novel diagnostic and prognostic biomarker in glioblastoma multiforme. Mol Cancer. 2018;17(1):74. 29558959 10.1186/s12943-018-0822-0 PMC5861620

[51] Min W, Dai D, Wang J, et al. Long Noncoding RNA miR210HG as a Potential Biomarker for the Diagnosis of Glioma. PloS One. 2016;11:e0160451. 27673330 10.1371/journal.pone.0160451 PMC5038942

[52] Shin Low S, Pan Y, Ji D, et al. Smartphone-based portable electrochemical biosensing system for detection of circulating microRNA-21 in saliva as a proof-of-concept. Sens Actuators B Chem. 2020;308:127718.

[53] How CW, Ong YS, Low SS, et al. How far have we explored fungi to fight cancer? Semin Cancer Biol. 2021. DOI:10.1016/j.semcancer.2021.03.009 33737109

[54] Chia WY, Kok H, Chew KW, et al. Can algae contribute to the war with Covid-19? Bioengineered. 2021;12(1):1226–1237. 33858291 10.1080/21655979.2021.1910432 PMC8806238

