# Supplementary figures and images for: Sevoflurane inhibits progression of glioma via regulating the HMMR antisense RNA 1/microRNA-7/cyclin dependent kinase 4 axis
Source: Bioengineered. 2021 Oct 30;12(1):7893–906. doi: 10.1080/21655979.2021.1976712 (PMC8806593; doi:10.1080/21655979.2021.1976712)

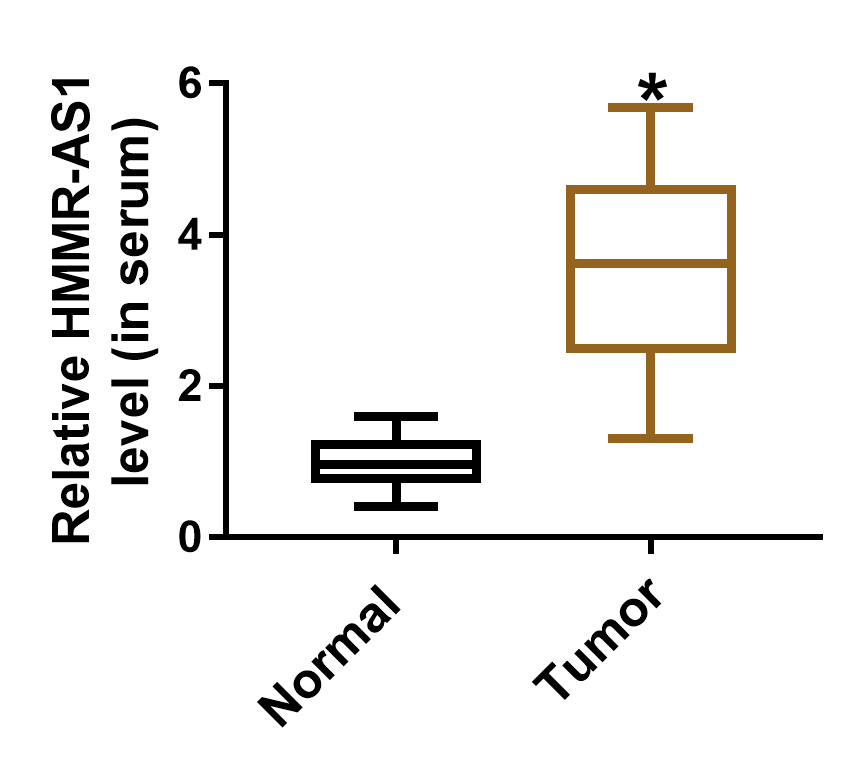

Supplement: Supplemental Material [file KBIE_A_1976712_SM8658.tif]
